# Prediction of heterogeneous differential genes by detecting outliers to a Gaussian tight cluster

**DOI:** 10.1186/1471-2105-14-81

**Published:** 2013-03-05

**Authors:** Zihua Yang, Zhengrong Yang

**Affiliations:** 1 Wolfson Institute for Preventive Medicine, Queen Mary University of London, Charterhouse Square, London EC1M 6BQ, UK; 2 College of Life and Environmental Sciences, Exeter University, Stocker Road, Exeter, EX4 4QD, UK

**Keywords:** Cancer, Outlier, Differentially expressed genes, Microarray

## Abstract

**Background:**

Heterogeneously and differentially expressed genes (hDEG) are a common phenomenon due to bio-logical diversity. A hDEG is often observed in gene expression experiments (with two experimental conditions) where it is highly expressed in a few experimental samples, or in drug trial experiments for cancer studies with drug resistance heterogeneity among the disease group. These highly expressed samples are called outliers. Accurate detection of outliers among hDEGs is then desirable for dis- ease diagnosis and effective drug design. The standard approach for detecting hDEGs is to choose the appropriate subset of outliers to represent the experimental group. However, existing methods typically overlook hDEGs with very few outliers.

**Results:**

We present in this paper a simple algorithm for detecting hDEGs by sequentially testing for potential outliers with respect to a tight cluster of non- outliers, among an ordered subset of the experimental samples. This avoids making any restrictive assumptions about how the outliers are distributed. We use simulated and real data to illustrate that the proposed algorithm achieves a good separation between the tight cluster of low expressions and the outliers for hDEGs.

**Conclusions:**

The proposed algorithm assesses each potential outlier in relation to the cluster of potential outliers without making explicit assumptions about the outlier distribution. Simulated examples and and breast cancer data sets are used to illustrate the suitability of the proposed algorithm for identifying hDEGs with small numbers of outliers.

## Background

A heterogeneously and differentially expressed gene (hDEG) is a gene which has an inconsistent expression pattern across its experimental samples. Typically, a large proportion of the experimental samples and the control samples form a tight cluster in low expressions. The remaining small proportion of experimental samples, namely the outliers, are observed to significantly deviate from the tight cluster towards high expressions. We use the word ‘tight’ to describe the cluster of null (or low) expressions of a hDEG as the null variance is typically small compared to the null-outlier distance. In situations where the few highly expressed outliers of a non-differential gene are caused by measurement error, it is also useful to distinguish such genes with hDEG characteristics. The existence of hDEGs has been established in various experiments ( [1-8]). Suppose we have the expressions of *m* genes. The standard *t* statistic under-estimates the significance in testing the difference across the control and experimental samples of a hDEG. COPA (cancer profile outlier analysis) [9] proposed modifying the Student *t* statistic to be a ratio of the distance between the *r*th (default 9th) percentile of experimental samples and the median of all samples over the median absolute distance (deviated from the whole sample median), *i.e.*,

(1) $$t_{i}^{\text{COPA}}=\frac{q_{r}(y_{i})-\lambda_{i}}{\sigma_{i}}\;i=1,\ldots ,m$$

where $\sigma_{i}=1.4826\times \text{med}(x_{i}-\lambda_{i},y_{i}-\lambda_{i})$, **x**_*i*_ and **y**_*i*_ represent control samples and experimental samples of the *i*th gene respectively, *q*_*r*_(**y**_*i*_) is the *r*th percentile of **y**_*i*_ and *λ*_*i*_ is the median of both **x**_*i*_ and **y**_*i*_. The quantile-median difference in (1) summarises the null-outlier distance using a single value of **y**_*i*_. To make outlier detection more efficient, the outlier-sum (OS) statistic [10] sums over outliers, $t_{i}^{\text{OS}}=\underset{j}{\sum }(y_{\text{ij}}-\lambda_{i})\sigma_{i}^{-1}$ where the outliers are defined as $\left\{y\in y_{i}:y>q_{75}(x_{i},y_{i})+\text{IQR}(x_{i},y_{i})\right\}$. Outlier robust *t* statistic (ORT) uses the same statistic but defines the outliers in relation to the control samples only $\left\{y\in y_{i}:y>q_{75}(x_{i})+\text{IQR}(x_{i})\right\}$[11]. Maximum ordered subset *t* statistic (MOST) defines the outliers to be the top *k* experimental samples and chooses *k* by optimising a normalised *t* statistic [12]. The least sum of ordered subset square *t* statistic (LSOSS) [13] also compares the controls with a subset of the top *k* experimental samples, $t_{i}^{\text{LSOSS}}=k({\bar{y}}_{i}^{\left(k\right)}-{\bar{x}}_{i})S_{i}^{-1}$ where ${\bar{x}}_{i}$ is the mean of control samples, ${\bar{y}}_{i}^{\left(k\right)}$ is the mean of top *k* experimental samples and *S*_*i*_ is the pooled standard deviation of the set of control samples plus non-outlier experimental samples and the set of outlier experimental samples. *k* is optimised iteratively to minimise the within-cluster variance. We propose a new algorithm for detecting hDEGs with a small number of outliers by detecting outliers via gap (DOG) maximisation. What makes this approach different from the existing methods is that we assess each potential outlier in relation to a tight cluster of non-outliers. This avoids modelling the highly expressed outliers explicitly. This is especially important when the number of outliers is small. The proposed algorithm classifies each gene as a hDEG or non-hDEG by locating potential outliers and summarises it using the average of the standardised outlier expressions. We will use simulated examples and a breast cancer dataset to illustrate the effectiveness of the proposed algorithm in detecting hDEGs with few outliers. We will also show how effective test algorithms are when varying conditions.

## Results and discussion

### Simulated examples

#### Scenario 1 - identification of a single hDEG

The algorithms are compared for the detection of a single hDEG with the number of outliers varied from one to nine. The results are summarised in Table 1. For a small number of outliers, COPA, MOST and LSOSS demonstrated relatively poor performances while DOG consistently gave significant *p*-values.

**Table 1:** Scenario 1

**Outlier no**	**COPA**	**OS**	**ORT**	**MOST**	**LSOSS**	**DOG**	**M**
1	0.656	0.141	0.115	0.328	0.439	0.011	1.00
2	0.489	0.028	0.035	0.255	0.153	0.001	2.00
3	0.420	0.004	0.008	0.148	0.101	0.001	2.99
4	0.504	0.002	0.002	0.171	0.093	0.001	4.00
5	0.523	0.0005	0.001	0.132	0.093	0.001	4.96
6	0.264	<10^−4^	0.0002	0.120	0.098	0.001	6.00
7	0.113	<10^−4^	<10^−4^	0.099	0.099	0.001	6.98
8	0.108	<10^−4^	<10^−4^	0.096	0.104	0.001	7.97
9	0.055	<10^−4^	<10^−4^	0.079	0.107	0.001	8.99

Scenario 1: average *p*-values for the simulated hDEG with variable outlier number from one to nine. M is the average number of outliers detected using DOG.

#### Scenario 2 - identification of multiple hDEGs (100 genes with 50 hDEGs)

**Figure 1:**
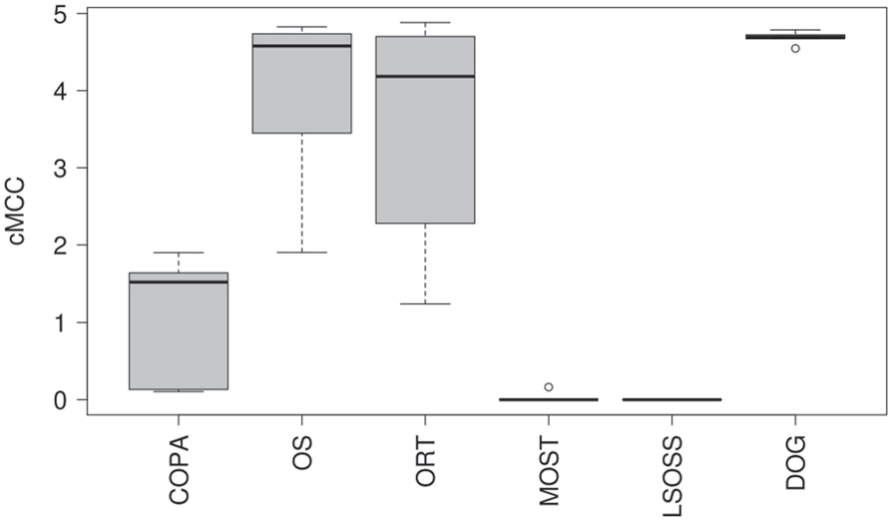
**cMCC.** Scenario 2: average cMCC of the six algorithms over (0, 0.01) for 1-5 numbers of outliers.

Over a critical *p*-value range from 0 to 0.01, DOG demonstrated the highest average cumulative Matthews correlation coefficient (cMCC, see Methods for more detail) across five sets of simulations with one to five outliers - Figure 1. Table 2 shows that DOG had very high classification rates compared with the other five algorithms. When the number of outliers exceeded two, OS, ORT and LSOSS gave more reasonable classification rates. COPA and MOST gave poor predictions overall.

**Table 2:** Scenario 2

**outlier no**	**COPA**	**OS**	**ORT**	**MOST**	**LSOSS**	**DOG**
1	0.54	0.77	0.72	0.51	0	1
2	0.55	0.9	0.92	0.51	0.55	0.99
3	0.66	0.94	0.96	0.57	0.93	0.99
4	0.73	0.95	0.99	0.73	0.99	0.99
5	0.69	0.93	0.95	0.73	1	1

Scenario 2: Total classification rates for the six algorithms in five simulations with 50 non-hDEGs and 50 hDEGs.

**Figure 2:**
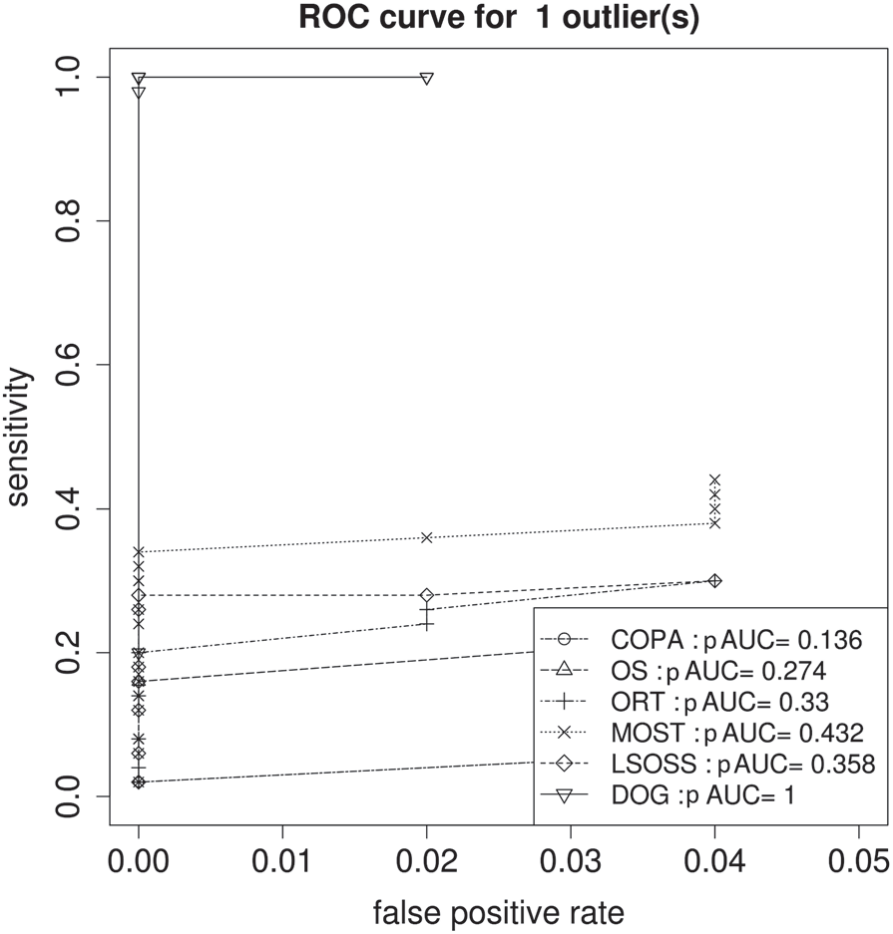
**ROC - one outlier.** Scenario 2: ROC curves of the six algorithms in detecting single outlier-hDEGs (in close up for low false positive rates).

**Figure 3:**
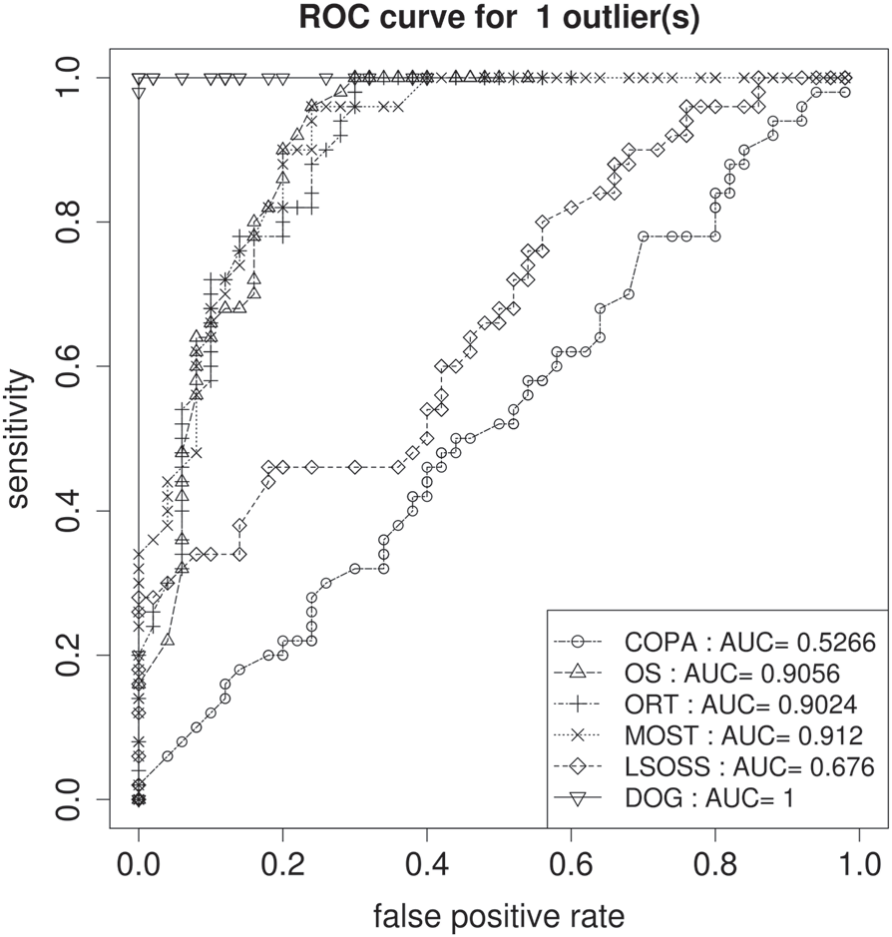
**ROC - one outlier.** Scenario 2: Full ROC curves of the six algorithms in detecting single outlier hDEGs.

 Figure 2 shows the ROC curves for the one-outlier simulations, it can be seen that DOG had a superior ROC curve with an partial AUC value of 1. Figure 3 illustrates the same ROC curves oover the complete range of false positive rate, COPA and LSOSS remained poor. We also found that as the number of outliers increased to five, most algorithms worked well with the exception of COPA. 

### Further simulated examples

We look at the sensitivitiy of DOG with respect to changes in certain assumptions and parameters.

#### Variable marginal null-outlier distance

We revisit the single-hDEG simulation but vary the marginal null-outlier distance (defined in Experimental design of Methods) from 0.5 to 2 with increments of 0.1 - Table 3. DOG’s *p*-values increased for a reduced marginal null-outlier distance but retained the most significant mean *p*-values for larger marginal null-outlier distances. MOST and LSOSS failed to detect the hDEG. DOG gave accurate estimates of the outlier number when the null-outlier distance was greater than one.

**Table 3:** Distance effect

***δ***	**COPA**	**OS**	**ORT**	**MOST**	**LSOSS**	**DOG**	**M**
0.5	0.6687	0.0283	0.0410	0.3634	0.1086	0.0497	0
0.6	0.6687	0.0258	0.0387	0.3278	0.1076	0.0495	0.03
0.7	0.6687	0.0236	0.0366	0.2918	0.1353	0.0472	0.38
0.8	0.6687	0.0220	0.0351	0.3566	0.1213	0.0421	0.92
0.9	0.6687	0.0204	0.0335	0.3418	0.1421	0.0340	1.37
1.0	0.6687	0.0187	0.0315	0.3171	0.1409	0.0271	1.75
1.1	0.6687	0.0170	0.0295	0.3005	0.1655	0.0198	1.85
1.2	0.6687	0.0157	0.0280	0.2863	0.1691	0.0157	1.92
1.3	0.6687	0.0140	0.0260	0.2807	0.1668	0.0117	1.98
1.4	0.6687	0.0125	0.0243	0.2964	0.1656	0.0083	1.99
1.5	0.6687	0.0117	0.0233	0.2875	0.2004	0.0066	2
1.6	0.6687	0.0103	0.0216	0.2820	0.1828	0.0045	2
1.7	0.6687	0.0094	0.0202	0.2656	0.1988	0.0032	1.99
1.8	0.6687	0.0089	0.0196	0.2658	0.1936	0.0028	2
1.9	0.6687	0.0078	0.0178	0.2699	0.2380	0.0018	2
2.0	0.6687	0.0072	0.0169	0.2563	0.2465	0.0012	2

Average *p*-values for single-hDEG simulations with two outliers and a varying distance, ***δ***, between non-outliers and outliers. M is the average number of outliers detected using DOG.

#### Non-Gaussian tight cluster

We simulated a Gaussian-mixture tight cluster (0.5 N(9,1)+0.5N(10,1)) to examine how DOG is affected by non-Gaussianity in the tight cluster. All other parameters were kept the same as those used in the single-hDEG simulation. The results were very similar to those seen previously - Table 4. In particular, the performances of COPA, OS and ORT have improved for the simulated non-Gaussian tight cluster.

**Table 4:** Non-Gaussian tight cluster

**Outlier no**	**COPA**	**OS**	**ORT**	**MOST**	**LSOSS**	**DOG**	**M**
1	0.2251	0.0156	0.0458	0.2847	0.5196	0.0031	0.99
2	0.0463	0.0120	0.0101	0.1692	0.2175	0.0015	1.99
3	0.0149	0.0017	0.0020	0.1492	0.1094	0.0020	2.96
4	0.0088	0.0003	0.0006	0.1270	0.0810	0.0014	3.99
5	0.0067	0.0001	0.0002	0.1062	0.0848	0.0015	4.97
6	0.0065	<10^−4^	<10^−4^	0.1045	0.0880	0.0015	5.94
7	0.0051	<10^−4^	<10^−4^	0.0887	0.0938	0.0013	6.96
8	0.0336	<10^−4^	<10^−4^	0.0828	0.0923	0.0014	7.92
9	0.0348	<10^−4^	<10^−4^	0.0821	0.0970	0.0012	8.98

Average *p*-values for the simulated hDEG with variable numbers of outliers for a mixture Gaussian ( 0.5N(9,1)+0.5N(10,1)) distributed tight cluster. M is the average number of outliers detected using DOG.

#### Control samples containing outliers

DOG can be modified to enable the detection of hDEGs when control samples contain outliers (see ‘’Allowing control samples to contain outliers of Methods. We illustrate this using the single-hDEG example with one outlier added to the control samples - Table 5. It can be seen that DOG accurately detected the outliers from both control and experimental samples. MOST and LSOSS failed to detect the hDEG.

**Table 5:** Control samples containing outlier

**Outlier no**	**COPA**	**OS**	**ORT**	**MOST**	**LSOSS**	**DOG**	**M**
1	0.2199	0.1167	0.1790	0.3165	0.4709	0.0009	2
2	0.1126	0.0509	0.0529	0.2327	0.3206	0.0009	3
3	0.1086	0.0095	0.0147	0.1942	0.2366	0.0008	4
4	0.1235	0.0017	0.0038	0.1468	0.1981	0.0008	5
5	0.0855	0.0001	0.0010	0.1358	0.2039	0.0006	6
6	0.0467	<10^−4^	0.0001	0.1225	0.1984	0.0006	6.99
7	0.0648	<10^−4^	<10^−4^	0.1105	0.2216	0.0006	8
8	0.0416	<10^−4^	<10^−4^	0.1016	0.2236	0.0006	9
9	0.0233	<10^−4^	<10^−4^	0.0872	0.2298	0.0007	9.99

Average *p*-values for single-hDEG simulations when control samples contain an outlier. The outlier number on the left column denotes the numer of outliers in experimental samples only. M is the average number of outliers in both control and experimental samples detected using DOG.

### Breast cancer data

**Figure 4:**
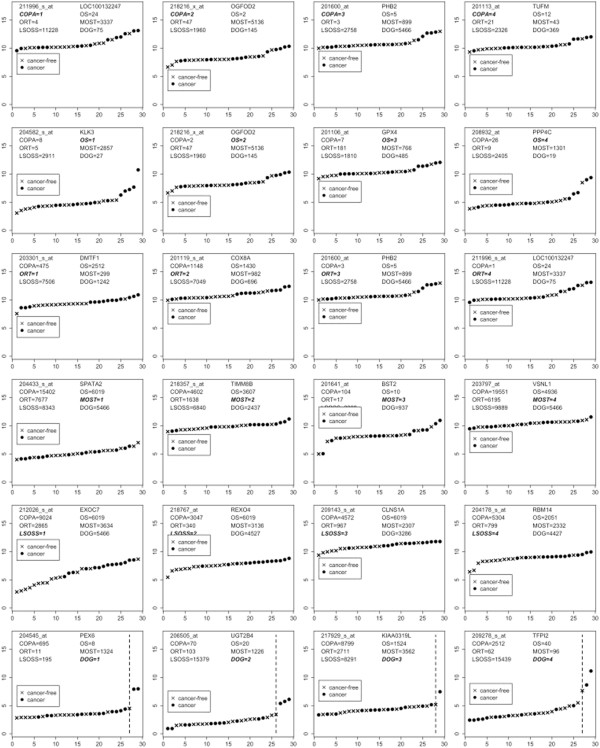
**COPA, OS, ORT, MOST, LSOSS, DOG.** Breast cancer data: log2 expressions of the top four hDEGs detected using COPA, OS, ORT, MOST, LSOSS, DOG. The vertical line indicates the separation of expressions in the tight cluster (left) and outliers (right).

**Figure 5:**
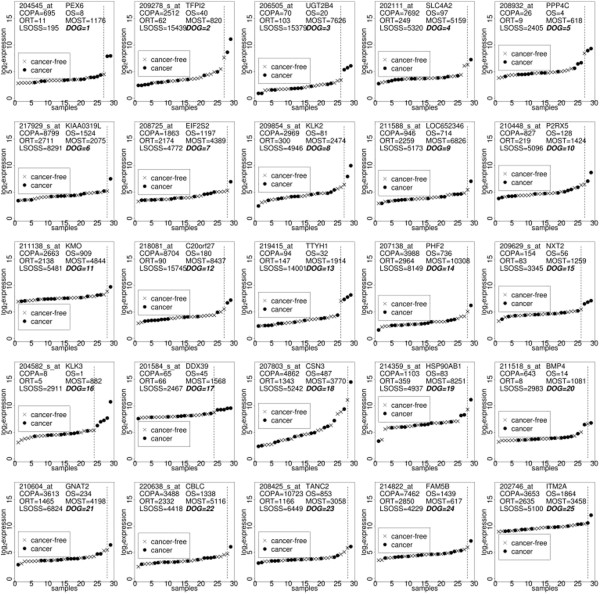
**DOG.** Breast cancer data: log2 expressions of the top 25 hDEGs detected using DOG.

Figure 4 illustrates the ordered expressions of the top four hDEGs as detected by the COPA, OS, ORT, MOST, LSOSS and DOG respectively (with annotations of rankings). The rankings of the genes were based on the order of the test statistics. The defining feature of DOG’s top four hDEGs, PEX6, TFP12, UGT2B4 and SLC4A2 (last row of Figure 4), is that they contain a few highly expressed outliers. Figure 5 shows the top 25 predictions of hDEGs using DOG for this data set. Existing literature have established these genes to be of biological relevance to the progression and treatment of breast cancer ( [14-23]).

**Figure 6:**
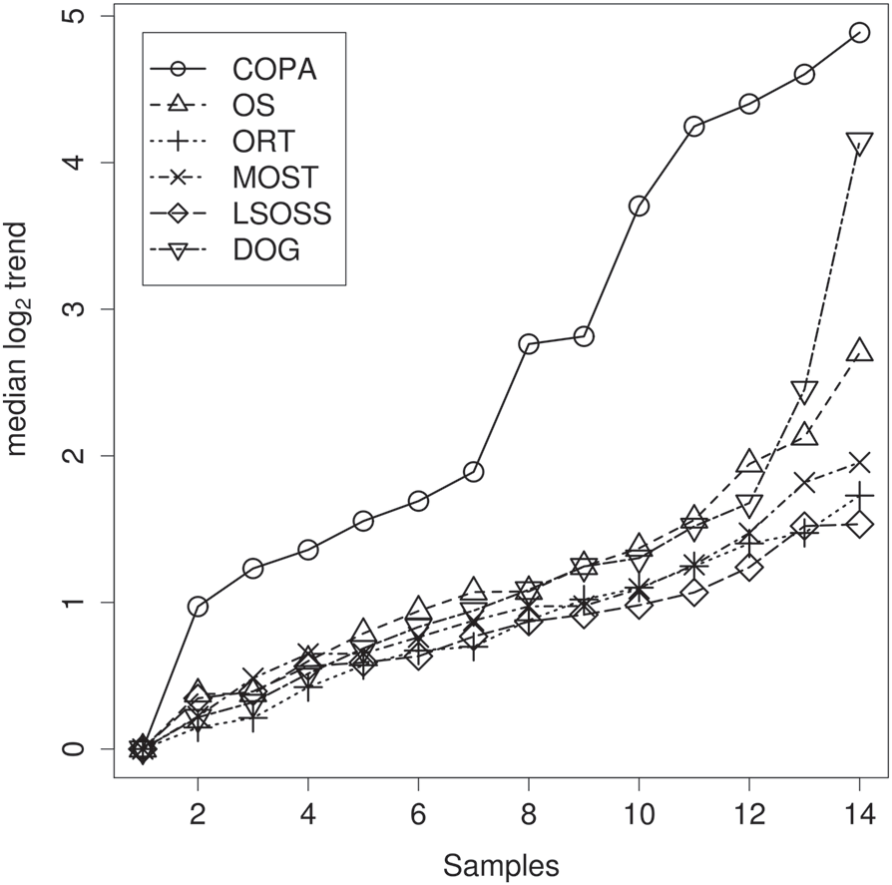
**Trends.** Breast cancer data: trends of scaled medians (median minus the minimum across each sample index) across the experimental samples of the log2 expressions of each algorithm’s unique top 100 hDEGs.

 Most other algorithms chose genes with a reasonably large pool of differentially expressed experimental samples expressed at a more moderate level. LSOSS also generally favoured ordinary DEGs. MOST chose a set of top four genes with only one or two moderately expressed outliers. Table 6 shows how the top 100 predictions of these algorithms overlap - COPA and OS are most similar in their rankings whilst DOG has a maximum of 15% overlap with OS. Using the ordered log2 expressions of each algorithm’s unique top 100 genes, Figure 6 illustrates the median expressions minus the minimum expressions for each experimental sample index. The unique top 100 genes for DOG and COPA showed the largest change across their experimental samples, their difference being that COPA favoured hDEGs with a larger number of outliers whilst DOG picked out hDEGs with small numbers of outliers. 

Using the significance analysis approach discussed in ‘’Significance analysis for real data of Methods, we estimated *p* values from sampling the replicates which then give us alternative *p* values based rankings of the genes. We also found the top four predictions ranked using the *p* values of DOG to be near identical to those ranked using its *t* statistics, though there were discrepancies in rankings for the lower ranking genes. Similar results were observed for the remainingfive algorithms.

## Conclusions

The difficulty in identifying hDEGs arises from the fact that only a small number of experimental samples are highly expressed at a much higher level than the non-outliers. As a result, various modified *t* tests target the subset of potential outliers which are then tested against the control group. In practice, for hDEGs with very few outliers, we found that these algorithms often identify hDEGs with insignificant deviations between the outliers and the tight cluster of non-outliers. Based on this observation, the proposed algorithm assesses each potential outlier in relation to the Gaussian tight cluster without making an explicit assumption about the outlier distribution. At each step, we update the posterior mean and variance of the tight cluster which are then used to evaluate the probability of an outlier being a random sample of the tight cluster. Examples of simulated and breast cancer data sets verify the suitability of the proposed algorithm in identifying hDEGs with small numbers of outliers. An extension of the algorithm which fully takes into account gene correlations will be presented in future work. For the breast cancer data, we found negligible correlations across the top ranking genes and very low correlations among the less significant genes.

**Table 6:** Ranking accordance

	**COPA**	**OS**	**ORT**	**MOST**	**LSOSS**	**DOG**
COPA		39.8	19.0	0.5	<0.1	9.3
OS			25.0	4.7	<0.1	14.9
ORT				3.6	2.5	11.1
MOST					0.5	2.0
LSOSS						<0.1

Breast cancer data: the overlap (percentage of accordant rankings) between top 100 predictions of the six algorithms.

## Methods

The proposed algorithm can be briefly summarised as follows. We first take the list of candidate outliers to be those experimental samples whose expressions are larger than the maximum expression of control samples. For the situation when control samples also contain outliers, see section ‘’Allowing control samples to contain outliers for a description of the necessary extension. The samples in the candidate list are sorted in an ascending order. The algorithm then updates the tight cluster of non-outliers by testing sequentially the samples in the updated candidate list of outliers. The test is terminated when a significant deviation between a candidate sample and the tight cluster is detected. We now give the steps in more statistical detail. First, let us introduce some notation. Let **x** denote the control samples and **y** the experimental samples of a gene or a probe set (we drop the gene subscript *i* for simplicity). The proposed DOG algorithm has the following steps: 

1. *Candidate outlier*: Given the union of **x** and **y**, **z**≡**x**∪**y**, we divide **z** into the candidate outlier set **z**^+^=*⇑*{*zj*+∈**z**|*zj*+> max(**x**)} and the non-outlier set $z_{j}^{-}=\{z_{j}^{-}\in z|z_{j}^{-}\leq \max(x)\}$ where *⇑* sorts the elements of a set in an ascending order.

2. *Detection*: Given a critical tail probability *α* and the corresponding threshold *t*_*α*_[24]. The first element in **z**^+^, $z_{1}^{+}$, is classified as the first outlier if

$$t=\frac{z_{1}^{+}-\mu }{\sigma }>t_{\alpha }$$

in which case the algorithm terminates and **z**^+^ is the set of outliers. We use a default value of *α*=0.05. The parameters *μ* and *σ*^2^ are posterior mean and posterior variance derived of the tight cluster. Details of estimating *μ* and *σ* are given below.

3. *Absorption*: On the other hand if *t*≤*t*_*α*_, we move *z*1+ to the tight cluster of non-outliers, **z**^−^←**z**^−^∪*z*1+ and **z**^+^←**z**^+^∖*z*1+.

4. *Estimating the parameters of the tight cluster*: The parameters *μ* and *β*=*σ*^−2^ are updated using iterative Bayesian learning, *i.e.*, by maximising the posterior probability [24]. Given z∼N(μ,1/β) with conjugate priors $\mu ∼N(\mu_{0},1/\sigma_{0}^{2})$ and *σ*^2^=1/*β*∼*IG*(*a*,*b*), the log-posterior is

(2) $$\begin{array}{c} \log P(\theta |z^{-},\alpha )\propto \log ℒ(z^{-}|\mu ,\sigma^{2})+\log\text{IG}(\sigma^{2}|a,b)\\ \;+\log N(\mu |\mu_{0}\sigma_{0}^{2}) \end{array}$$

where

$$\begin{array}{l} \log ℒ(z^{-}|\mu ,\sigma^{2})\propto \log\beta /2-\underset{z_{j}\in z^{-}}{\sum }\beta {\left(z_{j}-\mu \right)}^{2}/2\\ \log\text{IG}(\sigma^{2}|a,b)\propto a\log b+(a+1)\log\beta -\mathrm{b\beta}\\ \log N(\mu |\mu_{0},\sigma_{0}^{2})\propto -\sigma_{0}^{2}{\left(\mu -\mu_{0}\right)}^{2}/2 \end{array}$$

and *θ*=(*μ*,*β*) and $\alpha =(\mu_{0},\sigma_{0}^{2},a,b)$. Suppose *n* is the number of expressions in the tight cluster for the current iteration. For simplicity, we set *μ*_0_=*med*(**z**^−^), *a*=1, *b* is set to be the maximum variance of expressions calculated gene by gene. To simplify the notation, we let $\beta_{0}=\sigma_{0}^{-2}$. *β*_0_ is updated recursively but we set its initial value to be $\beta_{0}^{\left(1\right)}=0.1$. The *maximum a posteriori* probability procedure then gives the updates

$$\begin{array}{l} \mu =\frac{\beta \underset{j}{\sum }z_{j}+\beta_{0}\mu_{0}}{\mathrm{\beta n}+\beta_{0}};\;1/\beta =\frac{\underset{j}{\sum }{\left(z_{j}-\mu \right)}^{2}+2b}{n+2a+2};\\ z_{j}\in z^{-}\;1/\beta_{0}=\frac{{\left(\mu -\mu_{0}\right)}^{2}/2+b}{a+1}\;. \end{array}$$

Repeat 3 and 4 until the first outlier (with the lowest expression) is detected or until all candidate outliers have been classified as non-outliers.

5. *Classification*: A gene for which the set **z**^+^ is non-empty is classified as a hDEG.

The summary statistic for a gene is taken to be the average of the outlier statistics $\underset{j\in z^{+}}{\sum }t_{j}/|z^{+}|$. We use the average as opposed to the sum of outlier contributions as we prioritise the detection of hDEGs with few outliers.

### Remark 1

We allow the hyperparameters *μ*_0_ to be evaluated directly from the dataset. We set $\beta_{0}^{\left(1\right)}$ to be 0.1, *β*_0_ is then updated iteratively in the algorithm. We desire the tight cluster variance prior to be densely distributed around the small values, thus we choose *a*=1 and *b* to be the maximum gene sample variance. In practice, we found that a large *b* and a small *a*≤1 optimise detection rates.

### Remark 2

It is clear that for a finite replicate number, the difference in mean and variance of the tight cluster at two sequential steps are bounded. Asymptotically, as the sample size increases at each iteration, these differences converge toward zero since the posterior mean and variance converge toward the sample mean and variance and the tight cluster only absorbs probable null samples. This then guarantees asymptotic algorithmic convergence. Convergence of parameters in step 4 for each iteration follow from standard Bayesian results [25].

### Cumulative Matthews correlation coefficient

We compare COPA, OS, ORT, MOST and LSOSS using the cumulative Matthews correlation coefficient (cMCC) which is the area under Matthews correlation coefficient (MCC, [26,27]) in the interval $\left[0,p^{*}\right]$:

(3) $$\bar{\rho }=\int_{0}^{p^{*}}\rho_{p}\text{dp},$$

the MCC *ρ*_*p*_ is defined as:

$$\rho_{p}\;=\;\frac{TP_{p}\times TN_{p}-FP_{p}\times FN_{p}}{\sqrt{\left(TP_{p}\;+\;FP_{p})(TP_{p}\;+\;FN_{p})(TN_{p}+FP_{p})(TN_{p}+FN_{p}\right)}}$$

Here, *TP*_*p*_, *TN*_*p*_, *FP*_*p*_ and *FN*_*p*_ represent the numbers of true positives (true hDEGs), true negatives (true non-hDEGs), false positives and false negatives respectively. These four quantities are determined based on a pre-defined critical *p*-value, i.e. *p*∈(0,*p*^⋆^].

### Total classification accuracy

The total classification accuracy is defined as

(4) $$\frac{TN_{p}+TP_{p}}{TN_{p}+TF_{P}+TP_{p}+FP_{p}}$$

where *TP*_*p*_, *TN*_*p*_, *FP*_*p*_ and *FN*_*p*_ have been defined above.

### Receiver operating characteristic (ROC) analysis

Receiver Operating Characteristic (ROC) [28] analysis has been used widely in outlier detection [11-13] for evaluating a classification model when varying the classification threshold, thus it is a useful tool for analysing the robustness of a classifier. As the threshold varies, the sensitivity $\left(\frac{TP_{p}}{TP_{p}+FN_{p}}\right)$ and the false positive rate $\left(1-\frac{TN_{p}}{TN_{p}+FP_{p}}\right)$ change accordingly. The ROC curve is then generated by linking all the pairs of false positive rates and sensitivities corresponding to a set of thresholds. The ROC curve of a desirable classifier is close to the top-left corner. In particular, we limit the false positive rate to less and equal to 5% as rates above this correspond to critical *p* values that are too large to be of practical relevance. We also calculate the area under a ROC curve (AUC) for quantitative evaluation. A large AUC value of close to 1 indicates a good classifier. As we truncate the false positive rate at an upper limit of 5%, we scale the AUC by this limit so that the best possible partial AUC value is one.

### Allowing control samples to contain outliers

In order for DOG to detect hDEGs when outliers are present in control samples, we can modify it slightly. Rather than using $z_{j}^{-}=\{z_{j}^{-}\in z|z_{j}^{-}\leq \max(x)\}$ in the first step of the algorithm, we can use instead the *r*^*th*^ (default is 90^*th*^) percentile of the control samples as the separation between samples belonging to the tight cluster and candidate outliers. Suppose the 90^*th*^ percentile of the control samples is denoted by *ς*, the selection of **z***j*− now follows $z_{j}^{-}=\{z_{j}^{-}\in z|z_{j}^{-}\leq \varsigma \}$. In practice, the *r*th percentile can be specified subjectively by the modeller.

### Significance analysis for real data

Existing literature on algorithms such as COPA, OS and ORT typically omits statistical significance when analysing real data. Here we propose a simple method for significance analysis. We assume that control samples contain no outliers. For each algorithm, we create new control and experimental replicates of a gene under the null hypothesis by sampling with replacement from only the control expressions of that gene. This is repeated 100 times to augment the set of null control and experimental samples. The null *t* statistics are then calculated for all genes. The *p* value for each gene is then calculated as the proportion of null statistics across all genes that exceed its observed *t* statistic.

### Experimental design

We first look at two simulated scenarios for comparing the algorithms. For both scenarios, the tight cluster of control samples and non-outlier experimental samples are drawn randomly from a Gaussian distribution with a mean of ten and a standard deviation of one. Both control and experimental categories have 30 replicates. The outliers are generated by adding distances to the maximum expression of the tight cluster. The distances are called marginal null-outlier distances in that such a distance measures the gap between the tight cluster and the first outlier which is closest to the tight cluster. The marginal oull-outlier distances are sampled from a Gaussian distribution centered at two and with a standard deviation 0.2. Similar to examples seen in [10], we generate 10,000 non-DEGs which gives us 10,000 null *t* statistics and corresponding *p*-values for the hDEGs. This approach is applied to each algorithm. All simulations are repeated 100 times. In the first scenario, we evaluate the algorithms for a single hDEG. In addition, we vary the number of outliers from one to nine. In the second scenario, we generate 50 non-DEGs and 50 hDEGs and vary the number of outliers from one to five. We also look at extensions of the single-hDEG experiment for testing DOG with regard to deviations from the model assumptions. We then apply the algorithms to the histological breast cancer dataset (GDS3139 - [29]) which was downloaded from the gene expression omnibus (GEO, http://www.ncbi.nlm.nih.gov/geo). It contains 22,283 genes for 14 breast cancer patients and 15 non-cancer women. The age of non-cancer women was matched with that of cancer patients. For evaluation and comparison of algorithms, we use the cumulative Matthews correlation coefficient (cMCC) and the total classification accuracy (with a critical *p*-value threshold of 0.01). We also carry out receiver operating characteristic (ROC) analysis [28] for variable critical *p*-value thresholds. Details of cMCC and ROC analyses have been given above.

## Competing interests

Both authors declare that they have no competing interests.

## Authors’ contributions

ZRY and ZHY designed the algorithm. ZRY implemented the algorithm. ZHY analysed the algorithm on the conceived simulated examples. ZRY acquired the dataset from GEO and analysed the algorithm on the real dataset. ZRY and ZHY wrote the paper. Both authors read and approved the final manuscript.
